# A patient report scale research to access the symptom burden in patients with IgA nephropathy

**DOI:** 10.1038/s41598-024-59586-3

**Published:** 2024-09-27

**Authors:** Nan Yang, Jiyu Tang, Xiaodi Li, Dingcheng Li, Bin Zhu, Qiang He, Yuqun Zeng, Juan Jin

**Affiliations:** 1 Graduate School of Clinical Medicine, Jinzhou Medical University, Jinzhou, Liaoning China; 2 Urology and Nephrology Center, Department of Nephrology, Zhejiang Provincial People’s Hospital, Affiliated People’s Hospital, Hangzhou Medical College, Hangzhou, Zhejiang China; 3 Department of Electrical and Computer Engineering, The University of Texas at Dallas, Richardson, TX USA; 4 Search & Discovery Team, Coupang, Seattle, WA USA; 5 Department of Nephrology, The First Affiliated Hospital of Zhejiang Chinese Medical University (Zhejiang Provincial Hospital of Traditional Chinese Medicine), Hangzhou, Zhejiang China

**Keywords:** IgA nephropathy, Symptom burden, Patients, Scale, Assessment tool, Chronic kidney disease, Glomerular diseases, IgA nephropathy, Kidney diseases, Nephrology, Signs and symptoms

## Abstract

Patients diagnosed with IgA nephropathy (IgAN) commonly experience a substantial burden of symptoms encompassing both physical and psychological aspects. Presently, there's a dearth of standardized assessment tools to effectively gauge the extent of symptom burden in IgAN patients. Therefore, this study aims to devise an IgAN Symptom Assessment Tool that enables a comprehensive evaluation of patient symptom burden and their self-perceived severity. Employing a prospective observational design, this study conducted a survey among patients diagnosed with IgAN at a hospital in China. The research team formulated an IgAN Symptom Burden Assessment Scale and administered a questionnaire to gauge patient symptom burden. Severity assessment was conducted on a 5-point Likert scale, with higher scores indicating a more pronounced burden of symptoms. The finalized scale comprised 14 distinct symptom items, and the questionnaire survey garnered responses from 200 patients, achieving a 100% response rate. Statistical analysis unveiled that nearly all patients regarded these symptoms as prevalent and significantly impactful on their daily lives, resulting in a considerable burden. Notably, mild oliguria, moderate nasal congestion, bitter taste , throat discomfort, alongside severe manifestations such as muscle weakness, fatigue, and foamy urine, were frequently reported by patients. The findings underscore that a substantial proportion of IgAN patients grapple with a significant burden of symptoms, emphasizing the imperative for healthcare providers to prioritize symptom management and implement proactive measures to alleviate these challenges. This study presents an innovative tool tailored for evaluating symptom burden specifically in IgAN patients. Subsequent research should center on validating this tool within larger patient cohorts to optimize the efficacy of symptom management in this demographic.

## Introduction

IgA nephropathy (IgAN) stands as the most prevalent primary glomerular disease globally, representing a significant contributor to chronic kidney disease and kidney failure. Its estimated incidence surpasses 2.5 cases per 100,000 individuals, with East Asian countries exhibiting the highest prevalence^1,2^. Predominantly affecting the younger and middle-aged population, IgAN can present with a variety of clinical manifestations, spanning from foamy urine to visible hematuria, and in severe instances, it may rapidly progress towards kidney insufficiency. The insidious nature of kidney ailments often results in delayed diagnosis and subsequent treatment. Following an IgAN diagnosis, approximately 25% to 50% of patients gradually advance to kidney failure within 10 to 20 years, owing to the chronic and recurrent nature of the disease^2^. This imposes a substantial symptomatic burden on affected individuals.

Understanding the distinct characteristics of this disease is pivotal in alleviating the symptomatic burden on patients. However, the current body of research lacks comprehensive assessments of symptom burden in individuals diagnosed with IgAN. This study seeks to address this gap by developing an assessment tool expressly tailored to evaluate the symptomatic burden associated with IgAN. Implementation of this tool will empower healthcare professionals to effectively screen and evaluate suspected cases, facilitating timely interventions that can significantly enhance patients' quality of life and prognostic outcomes.

## Methods

### Study design, setting and participants

**Figure 1:**
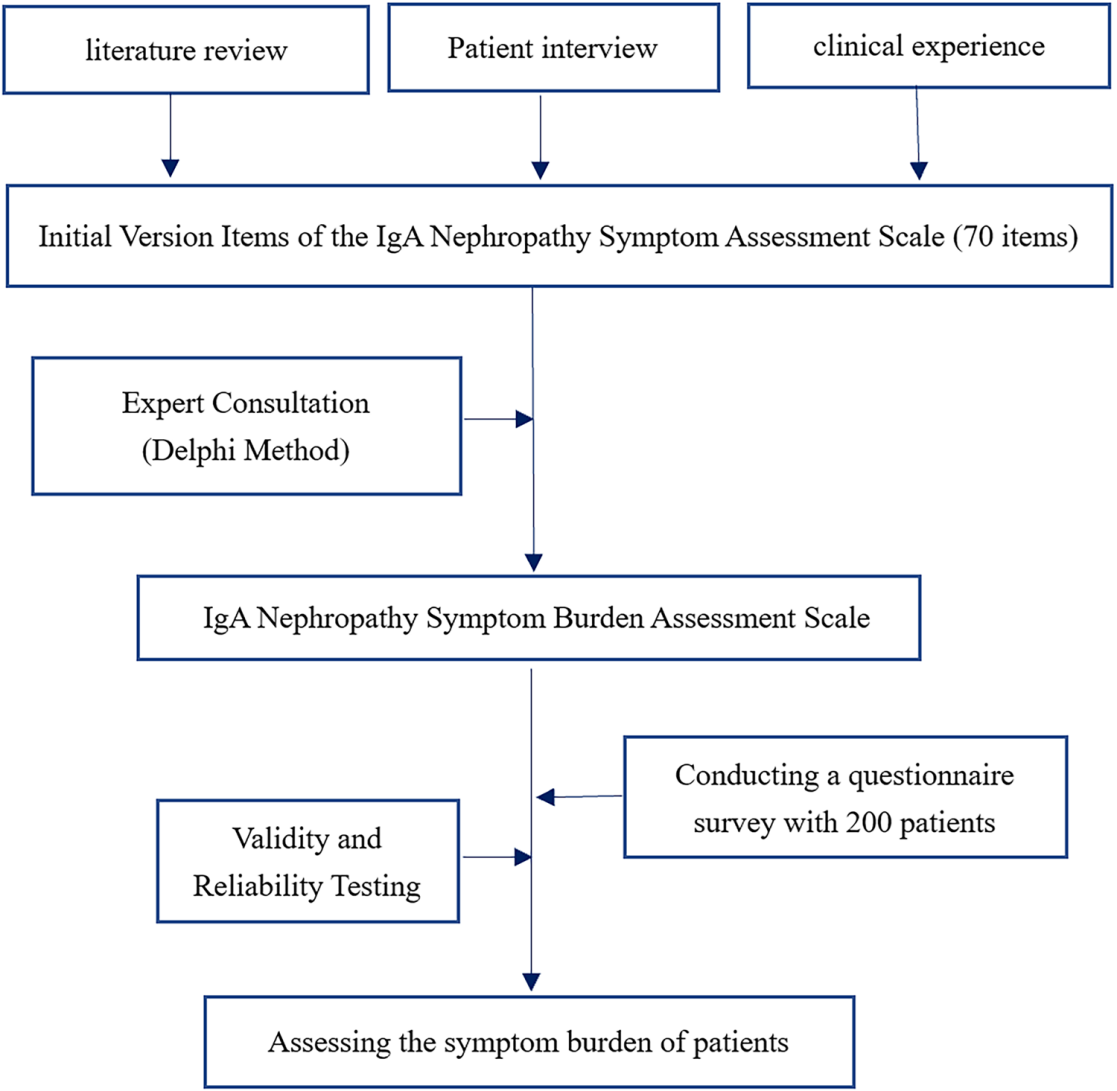
Flowchart of experimental design. In this study, we conducted a comprehensive process to assess the symptom burden of IgA nephropathy. Firstly, we conducted a literature review and extracted relevant information from domestic and international sources. Taking into account China's cultural context and clinical experience, we conducted questionnaire interviews with patients, generating a list of 70 items. Subsequently, we utilized the Delphi method with the participation of 54 experts to screen and refine these 70 items, resulting in the development of the IgA Nephropathy Symptom Burden Assessment Scale. This scale was then transformed into a questionnaire format. We distributed the questionnaire to 200 patients and collected data to evaluate the reliability and validity of the scale. Finally, based on the obtained data, we assessed the symptom burden experienced by these 200 patients.

The study conducted a formal questionnaire survey of patients with IgAN who sought outpatient or inpatient treatment at Zhejiang Provincial People's Hospital, from July 2022 to July 2023. This study has been approved by the Ethics Committee of Zhejiang Provincial People's Hospital (NO ZJPPHEC 2024I(038)). The inclusion criteria for this study involved patients diagnosed with IgAN confirmed through kidney biopsy and aged ≥ 18 years. Furthermore, participants were expected to have proficient communication skills, enabling them not only to complete surveys and communicate effectively with researchers but also to express their symptoms and discomfort accurately. Exclusion criteria encompass individuals with cognitive impairments or other conditions that impede participation, as well as patients diagnosed with malignant tumors. According to the DSM-V criteria, participants should not have acute or unmanaged psychiatric disorders^2^. Additionally, participants engaged in concurrent clinical trials where treatments could impact the study's outcomes were also excluded. All participants provided informed consent. The participating experts included nephrologists at a high level (associate professor or higher) or physicians with over five years of practical experience, and all experts volunteered to take part in this study and fulfilled the study requirements. The study flowchart was illustrated in Fig. 1.

### Design of the scale

#### Questionnaire structure

Drawing upon the validated Memorial Symptom Assessment Scale (MSAS)^3^, we employed this as the foundational model and tailored it through questionnaire interviews with patients. This iterative process yielded an initial pool of 70 symptom assessment items, covering diverse domains including the urinary, nervous, respiratory, digestive systems, overall symptoms, and psychological aspects (refer to supplementary table). Expert consultations utilizing the Delphi method ^4,5^refined this item pool, resulting in the final iteration of the IgAN symptom burden assessment scale. Quantitative assessment of patients' subjective discomfort utilized a 5-point Likert rating scale, with scores ranging from 0 to 4 signifying "Not at all severe", "Mildly severe", "Moderately severe", "Severe" and "Very severe", respectively. Higher scores denote increased symptom burden.

#### Expert questionnaire distribution

Using the expert consultation method^4^, nephrology specialists were engaged for evaluating the identified symptoms. The initial 70 items were condensed into an expert survey questionnaire, subjected to two rounds of consultations. After the first round, questionnaire items were based on expert feedback. Subsequently, a revised expert survey questionnaire was formulated, initiating the second round of consultations following a two-week interval.

The questionnaire comprised the following sections:

a). Expert Profile: Gathering essential information about each expert, including name, age, educational background, professional title, and years of experience.

b). Scale Item Pool: Experts assessed the relevance of each symptom item for inclusion in the assessment scale. They were encouraged to revise any inappropriate descriptions and suggest any overlooked items. Ratings were performed using a 5-point Likert scale, assessing the importance from 'not necessary at all' to 'extremely necessary', with scores ranging from 0 to 4. A comment section provided a platform for expert opinions on each item.

c). Expert Self-Evaluation of Judgment Criteria and Impact Level (*Ca*): Evaluating the expert's criteria for judgment and the influence level on the items based on theoretical analysis, practical experience, domestic and international literature, and intuitive judgment.

d). Expert Self-Evaluation of Familiarity with Items: Categorized familiarity levels included "unfamiliar", "not very familiar", "moderately familiar", "quite familiar", and "very familiar" with corresponding familiarity coefficients (*Cs*) of 0.1, 0.3, 0.5, 0.7, and 0.9, respectively.

e). Expert Additional Comments Form: Providing experts with a platform to share additional opinions or provide feedback.

The engagement of experts was assessed by the questionnaire response rate, while their level of expertise was represented by the expert authority coefficient (*Cr*), The *Cr* was directly proportional to both the *Ca* and *Cs* for each item. The formula used for its calculation:

$$Cr=\frac{Ca+Cs}{2}$$

which is assessed by experts according to their actual situation. Generally, the *Cr* ≥ 0.7 indicated that the experts had high authority in this research field, and the questionnaire had certain credibility^6^.

Incorporating insights from experts, revisions to scale items were made, involving appropriate additions, deletions, and modifications. Item importance was measured by the arithmetic mean of importance ratings, with a higher mean indicating greater item significance. The coefficient of variation (CV) represented variability in expert opinions regarding item importance, calculated as the ratio of the standard deviation to the mean, multiplied by 100%. A smaller CV indicated a lower degree of dispersion in expert opinions. The coordinated degree of expert opinion was expressed by *Kendall harmony* coefficient (*Kendall's W*). The value range of *Kendall's W* was 0–1, where a higher value of *W* indicated a better coordination degree of experts' opinions.

This study employed a criterion for item selection based on expert findings, considering items with a mean importance score > 3.0 and a CV < 0.20^6,7^.

#### Scale data collection

The final scale of the IgAN symptom questionnaire was employed in a survey conducted among IgAN patients meeting the inclusion criteria. Ensuring compliance with factor analysis requirements, the survey sample size maintained a ratio between each item and the sample size within the range of 1:5 to 1:10. Participants completed a demographic questionnaire encompassing age, gender, ethnicity, and marital status, alongside a self-assessment questionnaire on IgAN symptom burden. Standardized instructions were provided for self-completion or assisted completion of the questionnaires. On-site checks post-survey ensured completeness and validity, addressing any missing or incorrect entries. For test–retest reliability analysis, 30 patients were conveniently selected two weeks after the initial data collection.

### Statistical analysis

The data compilation was performed using Microsoft Excel 2010 software and meticulously verified by two individuals to ensure accuracy. IBM SPSS software for Windows, version 24.0 (IBM Corp) and AMOS 24.0 were utilized for statistical analysis. Descriptive statistics, including mean ± standard deviation, frequency, and composition ratio, were employed for quantitative data analysis. We initiated an "Item Analysis" to gauge the scale items' validity. This involved categorizing the data into high-scoring and low-scoring groups via the critical ratio (CR) method^8^. Subsequently, independent sample t-tests were employed to compare item scores between these groups. Items showing no statistically significant differences or having a CR value less than 3 were excluded.

The correlation coefficient method was employed for screening entries: items with a Pearson correlation coefficient (*r)* less than 0.4 with the total score were removed. *Cronbach's* α coefficient was used to assess scale reliability. If removing a specific item led to a substantial increase in the *Cronbach's* α coefficient, that item was considered for removal. During factor analysis, any item meeting criteria such as absolute factor loading < 0.4, communalities < 0.3, or improper factor attribution was considered for deletion.

Our assessment of the scale's feasibility involved reliability and validity tests. Reliability assessment encompassed internal consistency, split-half reliability, and test–retest reliability to evaluate stability and reliability. Validity tests included content validity, construct validity, discriminant validity, and convergent validity. Content validity was assessed via expert evaluations, utilizing the Item-level Content Validity Index (I-CVI) and Scale-level Content Validity Index (S-CVI). S-CVI calculations, comprising Universal Agreement S-CVI (S-CVI/UA) and Average S-CVI (S-CVI/Ave), aided in evaluating questionnaire item selection's rationale^9^. S-CVI/UA more than 0.80 and S-CVI/Ave more than 0.90 indicates excellent content validity^7^. Structural validity was evaluated using exploratory factor analysis (EFA) to understand the questionnaire's internal structure and item relationships in which by the Kaiser–Meyer–Olkin (KMO) test and Bartlett's test of sphericity. Discriminant and convergent validity were assessed via confirmatory factor analysis, ensuring accurate differentiation between concepts and overall measurement scale accuracy. The convergent validity was assessed by using Average Variance Extracted (AVE) and Composite Reliability. AVE value greater than 0.5 and Composite Reliability value greater than 0.6 indicated good convergent validity. If the square root of AVE is greater than the absolute value of the maximum inter-factor correlation coefficient, it expressed a good discriminant validity. Multiple fit indices were employed for the confirmatory factor analysis, including chi-square value (*χ*^*2*^), degrees of freedom (df), *χ*^*2*^/df ratio, root mean square error of approximation (RMSEA), comparative fit index (CFI), incremental fit index (IFI), normed fit index (NFI), Tucker-Lewis index (TLI), and relative fit index (RFI)^10^. These indices collectively evaluated the model's goodness of fit.

### Ethics approval

This study did not involve any form of human experimentation or manipulation of human samples. This study has been approved by the Ethics Committee of Zhejiang Provincial People's Hospital and has obtained informed consent from the patients. All methods were conducted in strict adherence to relevant guidelines and regulations.

## Results

### Patient demographics

We conducted a questionnaire survey among 200 patients with IgAN. The patients' average age was 40.19 ± 11.44 years, with females representing the majority (51.5%), and a significant proportion were married (82.0%). Of these, 152 patients (76.0%) identified their occupation as "clerical staff" and 192 patients (96.0%) hailed from southern regions. Additionally, 90 patients (45.0%) had concurrent hypertension. Table 1 summarized the demographic details of the surveyed patients.

**Table 1:** Demographic characteristics of 200 Patients with IgA nephropathy participating in the questionnaire survey.

Demographic	Descriptor	Value
Gender, n (%)	Female	103 (51.5)
	Male	97 (48.5)
Marital status, n (%)	Married	164 (82.0)
	Unmarried	36 (18.0)
Occupation, n (%)	Student	6 (3.0)
	Clerical staff	152 (76.0)
	Retired	6 (3.0)
	Farmer	7 (3.5)
	Others	29 (14.5)
Region, n (%)	South City	192 (96.0)
	North city	8 (4.0)
Age, n (%)	≤ 20	3 (1.5)
	21 ~ 40	108 (54.0)
	41 ~ 60	82 (41.0)
	≥ 61	7 (3.5)
Ethnic group, n (%)	Han Chinese	199 (99.5)
	Chinese Yi ethnic minority group	1 (0.5)
Presence of hypertension	Yes	90 (0.5)
	No	110 (0.6)

### Expert inquiry analysis

A set of expert inquiries were distributed to 54 eligible experts, and we conducted a comprehensive analysis of their demographic profiles. All 54 experts specialized in nephrology, with an average age of 49.96 ± 6.82 years and an average work experience of 27.02 ± 7.88 years. The majority were male (61.0%), and 51 experts (94.4%) held the position of associate chief physician or higher. Detailed demographic data can be found in Table 2.

**Table 2:** Demographic characteristics of Delphi panel (N = 54).

Demographic	Descriptor	Population (%)
Gender, n (%)	Female	21 (38.9)
	Male	33 (61.1)
Region, n (%)	Zhejiang Province	54 (100.0)
Age, n (%)	< 40	3 (5.6)
	40 ~ 50	28 (51.9)
	51 ~ 60	23 (42.5)
Years of clinical experience, n (%)	5 ~ 10	2 (3.7)
	11 ~ 20	6 (11.1)
	21 ~ 30	29 (53.7)
	> 30	17 (31.5)
Professional title, n (%)	Junior professional title	3 (5.6)
	Intermediate professional title	9 (16.6)
	Senior professional title	42 (77.8)
Specialty, n (%)	Nephrology	54 (100.0)

The experts' engagement was measured by the effective response rate of the questionnaires. In the initial questionnaire distribution, 54 were sent out and 54 valid responses were received, marking a 100% effective response rate—an indicator of their high level of proactivity. The *Cr* was 0.850, and the *Kendall’s W* was 0.327 (*P* < 0.001), signifying robust consistency and reliability in the survey results.

Following expert opinions, discussions within the project team, and considering the current scenario, items within the pool were merged, deleted, and modified. Notably, several items were merged for clarity and conciseness. For instance, various throat discomforts were unified under "throat discomfort", while diverse emotional indicators were consolidated into "depressive". Subsequently, 47 non-compliant items were removed, resulting in a revised scale comprising 14 items, as shown in Table 3.

**Table 3:** Symptom burden assessment scale for IgA nephropathy.

Symptom	The severity of the symptom burden				
	Not at all	Mild	Moderate	Severe	Very severe
	0	1	2	3	4
**Urinary system symptoms cluster**					
Foamy urine					
Oliguria					
Hematuria					
Nocturia					
**Pharyngeal symptoms cluster**					
Throat discomfort					
Nasal congestion					
Bitter taste					
**Physical symptoms cluster**					
Fatigue					
Muscle weakness					
Dropsy					
Lumbago					
Sexual issues					
**Psychological symptoms cluster**					
Anxiety					
Depressive					

The second round of questionnaires, sent two weeks after the initial distribution, garnered 54 valid responses, reflecting a 100% valid response rate. The *Cr* remained high at 0.850, with a *Kendall’s W* of 0.294 (*P* < 0.001), indicating unanimous agreement among the experts. Consequently, all 14 symptoms were retained based on this collective consensus.

### The scale analysis

This study rigorously scrutinized the scale's effectiveness through various analytical approaches, ensuring a comprehensive assessment of its items:

CR: The cohort of 200 patients with IgAN underwent a thorough symptom burden assessment. These individuals were categorized into high- and low-score groups based on their total scores. Impressively, all items demonstrated statistically significant differences (*P* < 0.05), underscoring their relevance. The CR values, ranging between 4.262 to 13.344 and surpassing the acceptable threshold (> 3), reinforced the retention of all items within the scale.

Correlation Coefficient: Establishing the relationship between individual symptoms and the overall score, Pearson correlation coefficients were computed. The majority of items exhibited robust correlations (ranging from 0.314 to 0.727, *P* < 0.05) except for the " hematuria " item, which displayed a coefficient slightly below the stipulated threshold of 0.4.

*Cronbach’s* α Coefficient: A critical evaluation of the scale's internal consistency led to an interesting finding. Upon exclusion of the " hematuria " item, the scale's *Cronbach's* α coefficient saw a noteworthy increase from 0.832 to 0.864. This implies that while the " hematuria " item weakened the scale's internal consistency, its exclusion bolstered its reliability.

Factor Analysis: Employing factor analysis, this study unveiled 4 distinct factors explaining a cumulative variance of 66.6%. All items displayed satisfactory factor loading values exceeding 0.4, indicating their relevance in contributing to the identified factors. Remarkably, no items were deemed unfit for retention based on this analysis.

Surprisingly, the " hematuria " item exhibited less desirable outcomes in correlation and internal consistency assessments. However, collaborative discussions involving experts and team members led to a crucial decision: retaining the " hematuria " item. The consensus was based on insights from clinical experiences and patient interviews, unanimously acknowledging its pivotal role in comprehensively evaluating IgAN symptoms.

Ultimately, these meticulous analyses, while presenting mixed findings for the " hematuria " item, converged on the unanimous decision to retain it. This decision shaped the final version of the scale, comprising 14 items, each contributing uniquely to comprehensively assess the spectrum of IgAN symptoms.

### The performance analysis of the scale

The assessment of the scale's effectiveness in capturing IgAN symptoms involved a thorough examination, encompassing various tests that provided a comprehensive understanding of its reliability, validity, and structural coherence:

Content Validity Analysis: The evaluation commenced with an in-depth scrutiny by experts in the field. I-CVI was systematically gauged, yielding values between 0.83 and 1.00, signifying a high level of agreement among experts regarding item relevance. However, while the Universal Agreement Scale-level Content Validity Index (S-CVI/UA) returned a score of 0.290, indicating potential disagreements among experts, the Scale-level Content Validity Index (S-CVI/Ave) fulfilled the established standard of 0.9 or above. S-CVI/UA is affected by the number of experts; it is generally difficult to reach 0.8 if the number of experts is large. Therefore, this indicator is allowed to be lower than 0.8^7^.

**Figure 2:**
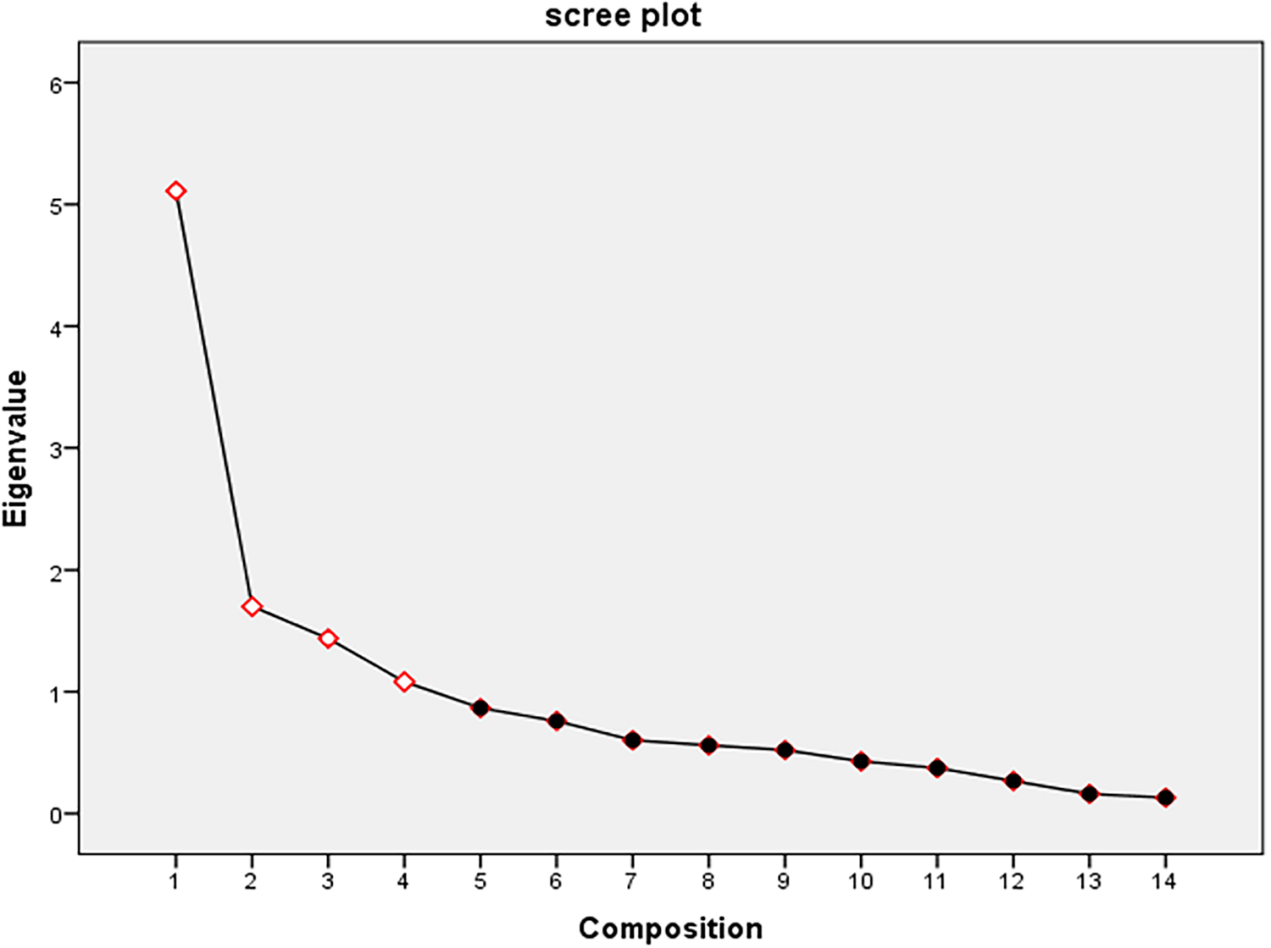
Scree plot of factor analysis for the scale.

Construct validity Analysis: EFA served as a pivotal tool for uncovering underlying structures within the dataset. EFA showed that The KMO value of the scale was 0.804, and the Bartlett's sphericity test was statistically significant (*χ*^*2*^ = 1271.15, *P* < 0.001), indicating that it is highly suitable for factor analysis^11^. Utilizing principal component factor analysis with varimax orthogonal rotation, the examination revealed the presence of four distinct factors that collectively elucidated 66.6% of the cumulative variance by identified four factors with eigenvalue > 1, showed in scree plot Fig. 2. This structural analysis highlighted specific item-factor relationships, showcasing loadings ranging from 0.536 to 0.887, As shown in Table 4. The identification of these factors ("Physical Symptoms Cluster", "Psychological Symptoms Cluster", "Pharyngeal Symptoms Cluster" and "Urinary System Symptoms Cluster") allowed for a deeper comprehension of the symptomatology and facilitated a structured understanding of symptom clusters related to IgAN.

**Table 4:** Factor loadings of symptom cluster assessment scale for IgA nephropathy patients (n = 200).

Symptom	Physical symptoms cluster	Psychological symptoms cluster	Pharyngeal symptoms cluster	Urinary system symptoms cluster
1. Muscle weakness	0.849			
2. Fatigue	0.764			
3. Dropsy	0.644			
4. Lumbago	0.637			
5. Sexual issues	0.609			
6. Depressive		0.878		
7. Anxiety		0.859		
8. Throat discomfort			0.887	
9. Nasal congestion			0.851	
10. Bitter taste			0.590	
11.Foamy urine				0.746
12. Nocturia				0.710
13. Oliguria				0.625
14. Hematuria				0.536
Factor eigenvalue	5.110	1.700	1.437	1.082
Cumulative variance contribution ratio (%)	20.980	36.777	52.351	66.642

The factor loading coefficients, the cumulative variance contribution ratio of each factor was showed. Factors named "Physical Symptoms Cluster", "Psychological Symptoms Cluster", "Pharyngeal Symptoms Cluster" and "Urinary System Symptoms Cluster".

Confirmatory Factor Analysis (CFA) Assessment: Validation of the identified factors was further reinforced through Confirmatory Factor Analysis. The model's fit indices, including *χ*^*2*^, RMSEA, CFI and other metrics, demonstrated favorable results, indicating a harmonious fit of the proposed model with the observed data. This analysis substantiated the consistency and validity of the identified factors, fortifying the scale's structural integrity.

Convergent and Discriminant Validity Evaluation: The assessment of convergent validity showcased the capacity of the scale to measure consistent constructs across different factors. AVE values exceeding 0.5 for most dimensions and Composite Reliability values surpassing the 0.6 threshold reinforced the scale's internal consistency and its ability to capture distinct facets of IgAN symptoms (Table 5). Discriminant validity, established through AVE square roots exceeding inter-factor correlations, underscored the scale's capability to differentiate between distinct symptom dimensions (Table 6)^12^.

**Table 5:** Convergent validity of the scale.

Symptom	AVE	Composite reliability
Urinary system symptoms cluster	0.348	0.652
Pharyngeal symptoms cluster	0.574	0.8
Physical symptoms cluster	0.501	0.825
Psychological symptoms cluster	0.855	0.922

*AVE* Average Variance Extracted.

**Table 6:** Discriminant validity of the scale.

	Urinary system symptoms cluster	Pharyngeal symptoms cluster	Physical symptoms cluster	Psychological symptoms cluster
Urinary system symptoms cluster	**0.348**	–	–	–
Pharyngeal symptoms cluster	0.347	**0.574**	–	–
Physical symptoms cluster	0.574	0.424	**0.501**	–
Psychological symptoms cluster	0.438	0.363	0.503	**0.855**

The inter-factor correlation coefficients were expressed. The values on the diagonal (bolded) represented the square root of AVE.

–The duplicate data had been omitted.

Reliability Analysis: A robust evaluation of the scale's reliability encompassed various indices. The Cronbach's α coefficient demonstrated commendable internal consistency (0.832), affirming strong correlations and reliability among items. Moreover, post-standardization, the coefficient elevated to 0.854, further solidifying the scale's reliability. Split-half reliability coefficients, alongside test–retest reliability coefficients, substantiated the scale's stability over time and its consistent measurement of IgAN symptoms, showed in Table 7.

**Table 7:** Reliability testing of the scale.

Reliability test	Total scale	Factor 1	Factor 2	Factor 3	Factor 4
*Cronbach's* α coefficient	0.832	0.823	0.921	0.788	0.627
Split-half reliability	0.640	0.744	0.921	0.622	0.576
Test–retest reliability	0.963	0.980	0.868	0.961	0.908

This multifaceted analysis culminated in a comprehensive understanding of the scale's structural, content, and measurement properties. The findings substantiated its reliability, validity, and capacity to effectively capture and categorize diverse IgAN symptoms across multiple dimensions.

### The distribution of symptom burden

The exploration into the symptomatology of IgAN in our study of 200 patients revealed a rich tapestry of manifestations in Table 8, segmenting into four distinctive categories: the "Physical Symptoms Cluster", "Psychological Symptoms Cluster", "Pharyngeal Symptoms Cluster" and "Urinary System Symptoms Cluster".

**Table 8:** Severity of perceived symptom burden in IgA nephropathy patients.

Symptom	n	Perceived severity of the symptom burden				
		Not at all	Mild	Moderate	Severe	Very severe
		n (%)	n (%)	n (%)	n (%)	n (%)
Muscle weakness	200	0 (0.0)	8 (4.0)	43 (21.5)	95 (47.5)	54 (27.0)
Fatigue	200	0 (0.0)	9 (4.5)	55 (27.5)	90 (45.0)	46 (23.0)
Dropsy	200	0 (0.0)	78 (39.0)	60 (30.0)	36 (18.0)	26 (13.0)
Lumbago	200	0 (0.0)	22 (11.0)	67 (33.5)	63 (31.5)	48 (24.0)
Sexual issues	200	0 (0.0)	40 (20.0)	74 (37.0)	51 (25.5)	35 (17.5)
Depressive	200	0 (0.0)	13 (6.5)	73 (36.5)	72 (36.0)	42 (21.0)
Anxiety	200	0 (0.0)	9 (4.5)	72 (36.0)	77 (38.5)	42 (21.0)
Throat discomfort	200	0 (0.0)	11 (5.5)	104 (52.0)	52 (26.0)	33 (16.5)
Nasal congestion	200	0 (0.0)	18 (9.0)	119 (59.5)	28 (14.0)	7 (3.5)
Bitter taste	200	0 (0.0)	18 (9.0)	101 (50.5)	65 (32.5)	16 (8.0)
Foamy urine	200	0 (0.0)	6 (3.0)	29 (14.5)	86 (43.0)	79 (39.5)
Nocturia	200	0 (0.0)	64 (32.0)	75 (37.5)	41 (20.5)	20 (10.0)
Oliguria	200	3 (1.5)	143 (71.5)	28 (14.0)	15 (7.5)	11 (5.5)
Hematuria	200	60 (30.0)	38 (19.0)	28 (14.0)	36 (18.0)	38 (19.0)

Interestingly, while a significant majority acknowledged experiencing the complete spectrum of 14 symptoms post-diagnosis, a notable segment, roughly 30.0%, hadn't encountered hematuria. Equally intriguing was the small 1.5% cohort that reported no experience of oliguria, signifying the broad spectrum of symptom occurrence within this patient population.

Amid this broad spectrum, several symptoms emerged as the most prominent. Oliguria stood out prominently, affecting 71.5% of patients, signaling a subtle but marked decline in kidney filtration and excretory functions. Additionally, the pharyngeal symptoms were striking, impacting 50.5% to 59.5% of patients with moderate bitter taste, nasal congestion, and throat discomfort, likely associated with abnormal immune system activation leading to pharyngitis.

Of particular concern were the significant reports of severe muscle weakness and fatigue, highlighted by 47.5% and 45.0% of patients respectively, substantially impairing their daily functionality. Intriguingly, foamy urine emerged as a noteworthy symptom, reported as "very severe" by 39.5% and "severe" by 43.0% of patients. It's intriguing to note that despite experiencing foamy urine before diagnosis, many patients didn't prioritize seeking medical attention specifically for this symptom, often focusing on other discomforts or anomalies discovered during routine medical assessments.

On the psychological front, patients exhibited considerable stress levels, with 57.0% reporting severe to very severe depressive symptoms and 59.5% expressing severe to very severe anxiety symptoms. The psychological burden was often linked to the prolonged illness, hormonal fluctuations, and the relentless progression of the disease, echoing the trends often observed among kidney disease patients, significantly impacting their overall quality of life.

Moreover, 45.0% of patients reported concurrent hypertension. Interestingly, symptoms typically associated with hypertension, such as "dizziness" and "headache," were intentionally omitted from the questionnaire. This deliberate exclusion indirectly accentuates the questionnaire's reliability in accurately assessing the symptom burden specifically relevant to IgAN patients, underscoring its validity in capturing disease-specific symptoms accurately.

This detailed insight into the diverse and intricate symptom landscape within IgAN patients significantly inform tailored treatment strategies. The nuanced understanding of the multifaceted symptom presentation underscores the necessity for comprehensive care plans, integrating precise management strategies targeting both the physical and psychological aspects of symptoms to enhance patient care and quality of life.

## Discussion

Symptoms in the context of diseases like IgAN are intricate and nuanced. They represent the subjective experiences of individuals grappling with an ailment, encapsulating an array of sensations that may be abnormal or unsettling. This subjective lens through which symptoms are experienced significantly contributes to what is termed "symptom burden." This burden extends beyond the mere manifestation of symptoms, impacting the affected individuals on various levels—physically, emotionally, and socially. Moreover, it has broader implications, influencing not just the individual but also exerting pressures on societal healthcare resources and systems.

Initially, the exploration of symptom burden primarily focused on cancer patients. However, recognizing the substantial impact of symptoms on the overall experience of chronic diseases, researchers expanded their scope to encompass diverse conditions, including cardiovascular issues, gastrointestinal disorders, chronic kidney diseases like IgAN, and beyond^13–15^. This expansion of focus is particularly crucial in conditions like IgAN, where symptoms might manifest subtly or be overshadowed, leading to an underestimation of the disease's true burden.

A notable aspect in the context of IgAN is the often subtle nature of its symptoms. This subtlety poses challenges in their recognition and acknowledgment within the healthcare landscape. Consequently, patients may find their symptoms overlooked during medical assessments, resulting in a heightened burden of the disease on their well-being. This realization prompted the need for a more patient-centric approach in healthcare services, culminating in the development of a specialized symptom evaluation scale for IgAN. This scale serves as a pivotal tool in assessing and understanding the multifaceted symptomatology experienced by IgAN patients, contributing significantly to more tailored and effective medical care.

Moreover, symptoms in end-stage kidney disease, such as those seen in IgAN, hold immense significance beyond their mere manifestation. They often serve as crucial indicators for initiating essential clinical interventions like dialysis. For instance, the occurrence of severe uremic symptoms that persist despite conventional medication may prompt the consideration of dialysis treatment. This underlines the pivotal role that symptoms play in not only diagnosing but also guiding the treatment trajectory of kidney diseases.

To ensure the reliability and validity of the IgAN symptom assessment scale, a meticulous approach was adopted, incorporating extensive literature reviews, in-depth patient interviews, stringent patient selection criteria, and rigorous item selection processes. The resulting scale emerged as a robust tool capable of objectively and comprehensively evaluating the spectrum of symptoms experienced by IgAN patients. Importantly, its user-friendly nature allows patients to complete it independently within a short timeframe, reinforcing its feasibility for practical clinical application.

The analysis of symptom burden in IgAN patients unearthed a range of reported experiences, from mild manifestations like oliguria, nasal congestion, bitter taste, and throat discomfort to more severe symptoms such as muscle weakness, fatigue, and foamy urine. These findings aligned with previous research, shedding light on the potential underlying pathophysiological mechanisms driving these symptoms. For instance, the decline in kidney function contributing to oliguria, immune system activation leading to throat-related symptoms, and kidney filtration abnormalities causing protein loss and associated weakness and fatigue were among the observed correlations.

Hematuria in our study is primarily characterized by a noticeable change in urine color, which can appear as red, amber, or cola-colored. Interestingly, despite being a hallmark symptom of IgAN, the reported incidence of hematuria is relatively low. This observation raises questions about the variability and perceptibility of this symptom, suggesting that its absence might lead to underreported cases or even instances of undiagnosed hematuria. This discrepancy emphasizes the need for a deeper understanding of how symptom presentation evolves over the course of IgAN and how patients perceive and report these symptoms. The statistical results indicate that "foamy urine" is a relatively typical symptom of IgAN, as we know the clinical manifestation of "proteinuria".

Moreover, contrary to expectations, the study did not find a significant association between disease staging and symptom burden. This challenges conventional assumptions that disease severity correlates directly with the burden of symptoms. Even in early stages or with ongoing treatments like dialysis, patients continued to grapple with significant symptom burden, highlighting a gap in managing these symptoms effectively.

Furthermore, the study acknowledged the influence of various external factors on symptom burden, including demographic, economic, and socio-cultural elements. Economic disparities, for instance, were noted to potentially exacerbate symptom burden due to limited access to quality healthcare^15^. However, this study's limitations, such as its confined geographical representation and sample size, necessitate future research endeavors to broaden the sample pool and investigate additional factors influencing symptom burden. Education level, in particular, emerged as a potential influential factor, with low educational attainment speculated to independently predict increased symptom burden^16^.

Despite the study's limitations, it serves as a significant milestone in comprehending the intricate symptom burden experienced by IgAN patients. It underscores the imperative for healthcare providers to integrate symptom management strategies alongside clinical treatments. The study's findings advocate for a holistic approach, recognizing symptoms as not merely clinical indicators but as pivotal aspects of patients' overall well-being.

In conclusion, this research provides a comprehensive overview of the substantial burden of disease symptoms experienced by IgAN patients. It calls for heightened attention from healthcare providers and caregivers to prioritize strategies that alleviate these symptoms. Furthermore, the study's future trajectory aims at extensive longitudinal investigations, aiming to unravel the evolving nature of IgAN symptoms, their determinants, and contributing factors. This extended research will lay the groundwork for evidence-based strategies aimed at effectively managing and reducing the burden of symptoms in IgAN patients, fostering improved quality of life and better healthcare outcomes.

## Supplementary Information

Supplementary Table 1.

## Data Availability

All data collected or analyzed in this study have been included in the published article and its supplementary information files.
