# Supplementary material for: A patient report scale research to access the symptom burden in patients with IgA nephropathy
Source: Sci Rep. 2024 Sep 27;14:22104. doi: 10.1038/s41598-024-59586-3 (PMC11437089; doi:10.1038/s41598-024-59586-3)
Supplement: Supplementary file 1 — Supplementary Table 1. [file 41598_2024_59586_MOESM1_ESM.docx]

| **symptom** | **Symptom burden severity score** | | | | |
| --- | --- | --- | --- | --- | --- |
|  | **not at all** | **mild** | **moderate** | **severe** | **very severe** |
|  | **0** | **1** | **2** | **3** | **4** |
| **Urinary symptoms cluster** |  | | | | |
| Frequent urination, urgency and pain |  |  |  |  |  |
| Foamy urine |  |  |  |  |  |
| Oliguria |  |  |  |  |  |
| Hydrouria |  |  |  |  |  |
| Hematuria |  |  |  |  |  |
| Nocturia |  |  |  |  |  |
| **Pharyngeal Symptoms Cluster** |  | | | | |
| Dry throat |  |  |  |  |  |
| Sore throat |  |  |  |  |  |
| Discomfort in the throat |  |  |  |  |  |
| Fever |  |  |  |  |  |
| Cough |  |  |  |  |  |
| Polypnea |  |  |  |  |  |
| Breathing difficulties |  |  |  |  |  |
| Chest distress |  |  |  |  |  |
| Nasal congestion |  |  |  |  |  |
| Bitter taste |  |  |  |  |  |
| Dry mouth |  |  |  |  |  |
| **digestive systems Symptoms Cluster** |  | | | | |
| Food taste change |  |  |  |  |  |
| Nausea |  |  |  |  |  |
| Vomit |  |  |  |  |  |
| Dental ulcer |  |  |  |  |  |
| Lack of appetite |  |  |  |  |  |
| Stomachache |  |  |  |  |  |
| Diarrhea |  |  |  |  |  |
| Abdominal distension |  |  |  |  |  |
| Constipation |  |  |  |  |  |
| **Neurological symptom cluster** |  | | | | |
| Dizziness |  |  |  |  |  |
| Headache |  |  |  |  |  |
| Difficulty concentrating |  |  |  |  |  |
| Amnesiac |  |  |  |  |  |
| Nervous, the heart is not secure |  |  |  |  |  |
| Have a nightmare |  |  |  |  |  |
| A drowsy feeling crept over |  |  |  |  |  |
| Have trouble falling asleep |  |  |  |  |  |
| Insomnia |  |  |  |  |  |
| Easy to wake |  |  |  |  |  |
| **Physical Symptoms Cluster** |  | | | | |
| Fatigue |  |  |  |  |  |
| Muscle weakness |  |  |  |  |  |
| Cold limbs |  |  |  |  |  |
| Eyelid or facial swelling |  |  |  |  |  |
| Numbness or tingling in the hands and feet |  |  |  |  |  |
| Lumbago |  |  |  |  |  |
| Muscular soreness |  |  |  |  |  |
| Arm or leg swelling |  |  |  |  |  |
| Feeling bloated |  |  |  |  |  |
| Weight reduction |  |  |  |  |  |
| Weight gain |  |  |  |  |  |
| Sweating/night sweats |  |  |  |  |  |
| Pale skin and mucosa |  |  |  |  |  |
| Feeling hot/cold in your body |  |  |  |  |  |
| Itchy skin |  |  |  |  |  |
| The skin of the body appeared cyanotic red yellow rash |  |  |  |  |  |
| Feeling of lack of energy |  |  |  |  |  |
| Sexual issues |  |  |  |  |  |
| Palpitations, heart palpitations |  |  |  |  |  |
| Baldness |  |  |  |  |  |
| Blurred vision |  |  |  |  |  |
| **Psychological symptoms cluster** |  | | | | |
| feel nervous |  |  |  |  |  |
| feeling sad |  |  |  |  |  |
| depressive |  |  |  |  |  |
| Worried |  |  |  |  |  |
| Anxiety |  |  |  |  |  |
| Feel irritable |  |  |  |  |  |
| Feel guilty |  |  |  |  |  |
| Feel fear |  |  |  |  |  |
| Feel inferior |  |  |  |  |  |
| feel lonely |  |  |  |  |  |
| I don't feel like myself |  |  |  |  |  |
| Having serious physical problems |  |  |  |  |  |
| Feeling helpless |  |  |  |  |  |
